# Knowledge graphs of ethical concerns of cerebral organoids

**DOI:** 10.1111/cpr.13239

**Published:** 2022-05-17

**Authors:** Lulu Ding, Zhenyu Xiao, Xia Gong, Yaojin Peng

**Affiliations:** ^1^ Institute of Zoology Chinese Academy of Sciences Beijing China; ^2^ School of Life Science Beijing Institute of Technology Beijing China; ^3^ Beijing Institute for Stem Cell and Regenerative Medicine Beijing China; ^4^ University of Chinese Academy of Sciences Beijing China

## Abstract

**Objectives:**

The rapid development of cerebral organoid technology and the gradual maturity of cerebral organoids highlight the necessity of foresighted research on relevant ethical concerns. We employed knowledge graphs and conducted statistical analysis with CiteSpace for a comprehensive analysis of the status quo of the research on the ethical concerns of cerebral organoids from a bibliometric perspective.

**Materials and Methods:**

We performed a statistical analysis of published papers on cerebral organoid ethics, keyword co‐occurrence graph, literature co‐citation and knowledge clustering graph to examine the status of the ethics research, internal relationship between technological development and ethical research, and ethical concerns of the academia. Finally, we used a keyword time zone graph and related statistics to analyze and predict the trends and popular topics of future cerebral organoids ethics research.

**Results:**

We demonstrated that although the ethical concerns of cerebral organoids have long been discussed, it was not until 2017 that the ethical issues began to receive more attention, when cerebral organoids were gradually mimicking the human brain more closely and increasingly being combined with chimera research. The recent key ethical concerns are primarily divided into three categories: concerns that are common in life sciences, specific to cerebral organoids, and present in cross‐fields. These increasing ethical concerns are inherently related to the continual development of technology. The analysis pointed out that future research should focus on the ethical concerns of consciousness that are unique to cerebral organoids, ethical concerns of cross‐fields, and construction and improvement of legislative and regulatory systems.

**Conclusions:**

Although research on cerebral organoids can benefit the biomedicine field, the relevant ethical concerns are significant and have received increasing attention, which are inherently related to the continual development of technology. Future studies in ethics regarding cerebral organoid research should focus on the ethical concerns of consciousness, and cross‐fields, as well as the improvement of regulatory systems.

## Graphical abstract

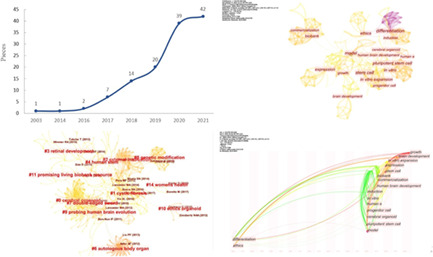

We performed a statistical analysis of published papers on cerebral organoid ethics, keyword co‐occurrence graph, literature co‐citation and knowledge clustering graph to examine the status of the ethics research. We also used a keyword time zone graph and related statistics to analyse and predict the trends and popular topics of future cerebral organoids ethics research.

## 1 INTRODUCTION

Advancements in stem cell‐derived human cerebral organoids in the form of in vitro three‐dimensional (3D) organoid cultures provide unprecedented opportunities for enhanced understanding of human brain development, drug screening and disease modelling studies of Alzheimer disease, autism spectrum disorder, neuropsychiatric diseases and adult central nervous system diseases (e.g., motor neuron disease and Parkinson disease). Moreover, they represent a unique virus exposure platform that enables improved understanding of the genesis of congenital brain abnormalities, particularly microcephaly, which is caused by Zika virus infection during early pregnancy.^1^, ^2^, ^3^, ^4^, ^5^, ^6^, ^7^ However, various ethical concerns accompany the advancement of cerebral organoid technology. Therefore, predicting and conducting research on the ethical concerns in this field are essential for governance and the healthy development of cerebral organoid technology. In spite of related research that has been conducted, such as cerebral organoids may arise ‘yuck factor’,^8^ the excessive emphasis on its technical rationality^9^ and the possibility of creating sentient cerebral organoids that could have a moral status,^10^ there are few papers on this issue. This study provides a bibliometric research on the ethical concerns of cerebral organoids to blaze a way in future relevant research at home and abroad.

On the basis of the literature on the ethical concerns of cerebral organoid in the Web of Science (WoS) database, this study used CiteSpace to conduct a knowledge graph analysis, determine the status of research on the ethics of cerebral organoid and explore the trends and patterns of such research. Potential research hotspots concerning the ethical problems of future cerebral organoid research were also analysed and anticipated.

## 2 MATERIALS AND METHODS

The materials used for analysis in this study were obtained from the WoS database. The search terms selected were ‘cerebral organoids ethics’, ‘organoids ethics’, ‘cerebral organoids ethical and ‘organoids ethical’. The search formula was ‘TS = (Cerebral organoids ethics) OR TS = (organoids ethics) OR TS = (cerebral organoids ethical) OR TS = (organoids ethical)’. The time span ranged from 2000 to 2021, the language was all languages, the document type was all document types and the retrieval time was 5 November 2021. A total of 126 documents were ultimately obtained.

The software CiteSpace (Ver. 5.8.R3c), which runs in the Java 8 environment, was employed for analysis. The documents obtained from the WoS database were used as the analysis object, and bibliometric analysis was conducted through a clustering algorithm. The specific operations and steps were as follows: (1) time slicing was performed on the literature from 2000 to 2021, and the analysis year value was set to 1 (every year); (2) the analysis object of the selection criteria was the first 20 pieces of literature (T20) published each year; (3) the node types were fixed; (4) the pruning algorithm (Pathfinder) in the streamlined function unit was used to prune and clarify the graph; and (5) for cluster selection, the clustering algorithm was used to process the citation network of knowledge clustering on the graph, and the label size and background colour of the nodes, lines and cluster names were adjusted.

## 3 TRENDS IN ETHICS RESEARCH

**FIGURE 1:**
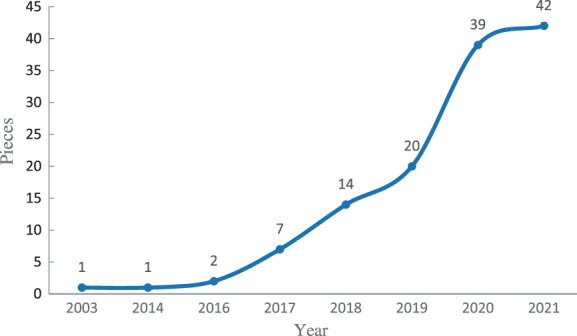
Statistics on the number of articles regarding research on ethical concerns of cerebral organoids

The number of relevant articles in the WoS database from 2000 to 2021 is presented in Figure 1. The number of annual publications is a direct reflection of research developments and trends. As illustrated in Figure 1, the number of articles published between 2003 and 2016 was small, but the number has substantially increased since 2017, especially from 2019 to 2020. Arguably, the expansion of the research on the ethical concerns of cerebral organoids may be caused by technological breakthroughs since 2016. The trend of the curve in Figure 1 indicates that the research on ethical concerns of cerebral organoids will continue to receive more attention from the academic community.

## 4 KEYWORD CO‐OCCURRENCE GRAPH ANALYSIS OF ETHICS RESEARCH

**FIGURE 2:**
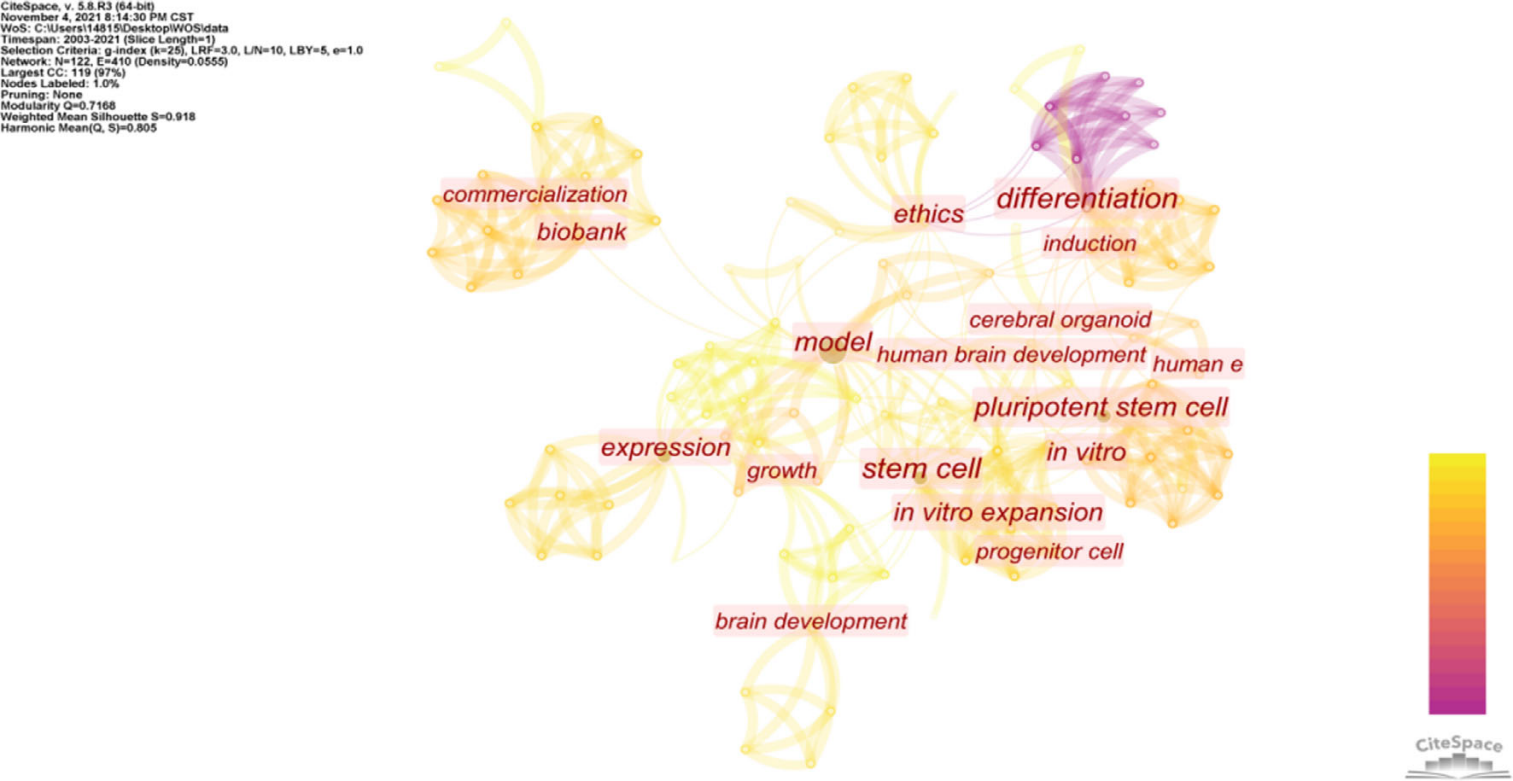
Keyword co‐occurrence graph of research on ethical concerns of cerebral organoids

The research hotspots of ethical concerns in the cerebral organoid field were presented through a keyword co‐occurrence graph, which was created through a keyword co‐occurrence analysis of the selected documents under the time slice and keyword threshold parameters of 1 and 9, respectively (Figure 2). The size of the circle in Figure 2 indicates the frequency of the keywords, and the thickness of the connection lines indicates the co‐occurrence of the relationship between each keyword. A thicker connection line between each keyword represents a closer relationship. More connections indicate higher centrality, which means the keyword hub function is strong and that the keyword is more likely to become a common research topic in the field. A total of 122 theme nodes and 410 network connections with a node density of 0.0555 were observed.

The PageRank index in Table 1 indicates the relevance and value of the keywords. A keyword that is connected to many other keywords has a high PageRank index and value. As presented in Table 1, keywords such as ‘model’, ‘stem cell’ and ‘ethics’ had higher frequencies and centralities than other keywords. ‘Ethics’, the first keyword that appeared, had a frequency of 6 (ranking third), centrality of 0.42 (ranking third) and PageRank index of 2.38 (ranking fourth). Therefore, of all the indicators, ‘ethics’ had a high ranking. Because it appeared first, it served as a vital link that closely connected other keywords and was thus deemed the centre and hotspot of research. Additionally, the first appearance of the keyword ‘pluripotent stem cell’ in 2017 and its high frequency (count = 6) implied that a technical breakthrough may have occurred in 2016 or 2017 in the construction of cerebral organoids by using pluripotent stem cells that subsequently invited additional ethical concerns.

**TABLE 1:** Statistics of popular keywords on the research of ethical concerns of cerebral organoids

No	Count	Centrality	Year^a^	PageRank index	Keywords
1	10	0.67	2017	2.7	Model
2	7	0.52	2019	1.91	Stem cell
3	6	0.42	2003	2.38	Ethics
4	6	0.16	2017	1.75	Pluripotent stem cell
5	4	0.26	2019	2.61	Expression
6	4	0.12	2018	1.51	Human brain development
7	4	0.33	2003	3.27	Differentiation
8	4	0.14	2017	1.61	Cerebral organoid
9	3	0.08	2017	1.49	Progenitor cell
10	3	0.14	2019	0.99	Cell

^a^ The year of first appearance.

## 5 ANALYSIS BASED ON KNOWLEDGE CLUSTERING

**FIGURE 3:**
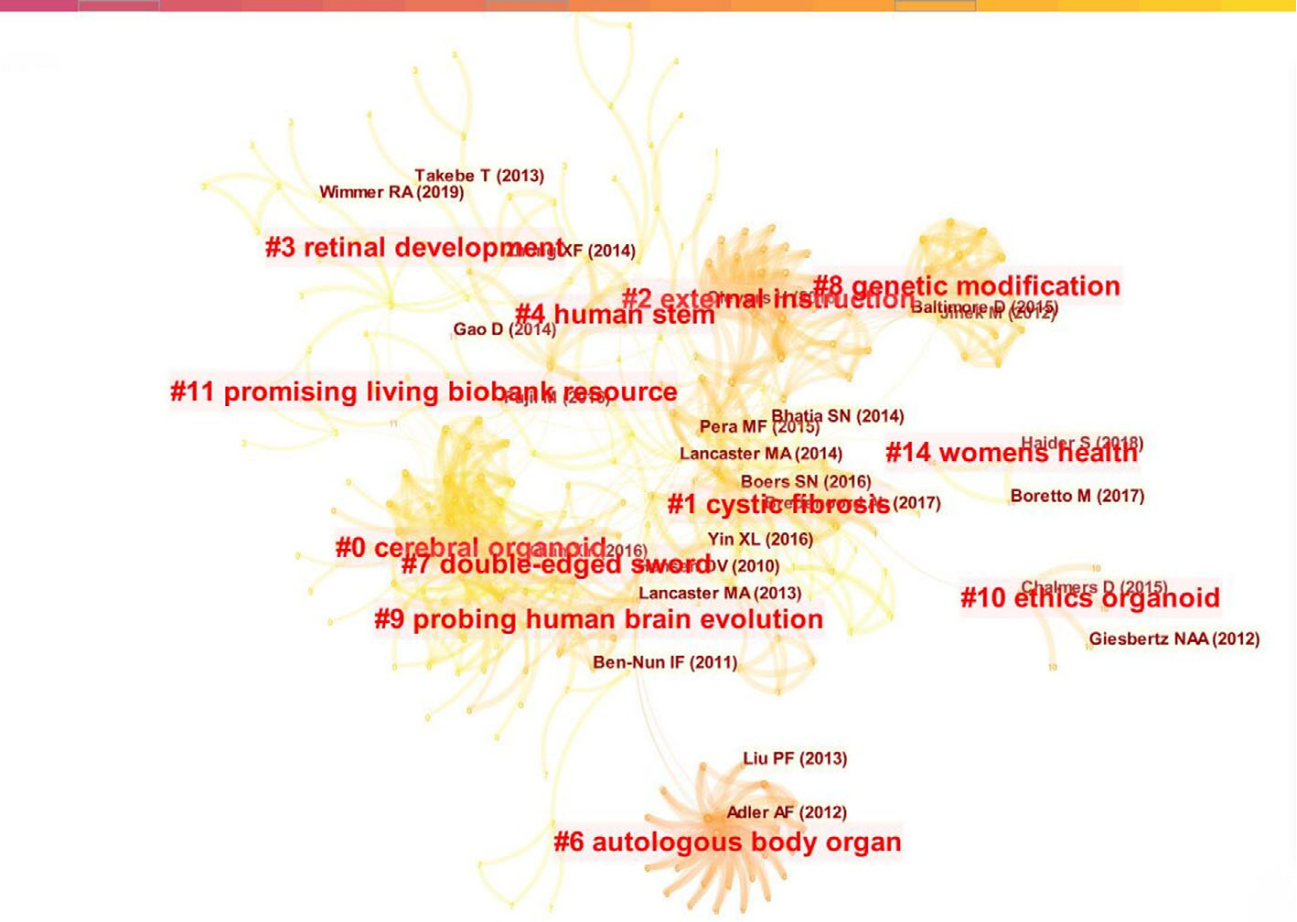
Literature co‐citation and knowledge clustering graph of research on ethical concerns of cerebral organoids

We conducted a knowledge clustering procedure based on the literature, and as presented in Figure 3, 12 knowledge clusters comprising 2 primary topics were obtained. Clusters 0, 3, 4, 6, 9, 11 and 14 pertained to cerebral organoid technologies, and clusters 1, 2, 7, 8 and 10 were related to the ethical concerns of cerebral organoids. The development of research on the ethical concerns in this field is inseparable from technological advancements. Thus, although the search terms selected for retrieval were phrases related to the ethical concerns of cerebral organoids, many cerebral organoid technology‐related articles in the generated knowledge graph had a primary theme. The ethical concerns of cerebral organoids can be further divided into three categories, concerns that are common in life sciences, specific to cerebral organoids and present in cross‐fields.

### 5.1 Common ethical concerns in life sciences

As observed in other disruptive technologies, the research and application of cerebral organoids, as a frontier in life science, may invite multifaceted ethical concerns. Individuals may resist the creation of stem cell‐based cerebral organoids based on ‘instinctive hostility’,^8^ and using cerebral organoids as a pathological modelling method also raises uncertainties about the instrumentalization of humans and whether this is the ultimate evolution of instrumental rationality.^9^ The use of cerebral organoids may undermine human dignity^10^, ^11^ and subvert some individuals' cognition of the origin of mankind, both in scientific and religious fields.^12^

In addition to these conceptual ethical concerns, cerebral organoids have generated practical ethical controversies regarding rights protection and supervision. Rights protection concerns the right to know and supervise the donors, recipients, researchers and ethical review agencies^13^, ^14^, ^15^, ^16^, ^17^, ^18^ and protect the privacy of the donors, patients and volunteers.^19^, ^20^ Additionally, when this technology matures, critical, sensitive decisions must be made about its use for medical resources or allocation to ordinary people,^13^ especially donors and recipients.^21^ Concerns about the welfare, rights and dignity of nonhuman primates^22^ and other laboratory animals^23^, ^24^ used in the experiments related to cerebral organoids also exist.

Regarding supervision, debates have occurred over the application scope of cerebral organoids. Scholars have launched discussions on the ethical and legal concerns of cerebral organoid use for laboratory research,^25^ clinical trials,^26^ commercial use^18^ and biobank development.^20^ Additionally, the ownership of human cerebral organoids is unclear and should be specified.^27^ The biosafety concerns caused by the accidental misuse of cerebral organoids should be prevented in advance.^28^ For example, human cerebral organoids could become infected with viruses due to negligence, and cross‐species virus infections could occur when chimeras are transplanted. Moreover, dishonest press coverage and misleading literature, films and television works that could affect public opinion should be managed in a timely manner.^29^ In short, improved rules related to regulation and supervision can ensure the reliable development of cerebral organoid technology.^30^

### 5.2 Ethical concerns specific to cerebral organoids

Human cerebral organoids are involved in simulating the human brain and may exhibit consciousness; thus, ethical concerns are abundant in the relevant research. The size of human cerebral organoids is small, their functions remain at a primitive stage, and the possibility of them generating consciousness is quite low.

However, with technological advancements, human cerebral organoids are likely to perceive neuronal stimulation (e.g., light and pain) and develop cognition and self‐awareness. In such an event, the due moral status (i.e., dignity, rights, and welfare) of human cerebral organoids would become a realistic ethical concern.^15^, ^31^, ^32^, ^33^ Moreover, scientists have injected immature neurons (preneural cells) derived from human embryonic stem cells into patients' brains for treatment; therefore, cerebral organoids may be used for transplantation in the future. Then, questions of whether patients would gain the personal characteristics, emotions or memories of the stem cell donors and whether they would suffer from self‐identity confusion or abnormal social cognition about their identities would become sensitive ethical concerns.^13^ Additionally, advancements in cerebral organoid technologies may enable the resurrection of dead brains, causing concerns over changing the standards of brain death and triggering debates about the nature of death and the identity of patients.^34^, ^35^

### 5.3 Ethical concerns in cross‐fields

With the evolution of the relevant research, cerebral organoids could be paired with living or inanimate systems in vitro to form an entity with human characteristics.^19^ This may lead to ethical concerns related to brain–computer interfaces. Cerebral organoids have been used in research on chimeras and will thus be subject to the ethical disputes that chimeras have encountered. For example, when human cerebral organoids are transplanted into a nonhuman primate host^36^ or other laboratory animals,^37^ a series of human–animal chimeras will be generated and obscure the boundary between humans and animals.^38^, ^39^ The corresponding dignity, rights and welfare of such chimeras are pressing ethical concerns that must be discussed.

## 6 KEYWORD TIME ZONE GRAPH ANALYSIS

**FIGURE 4:**
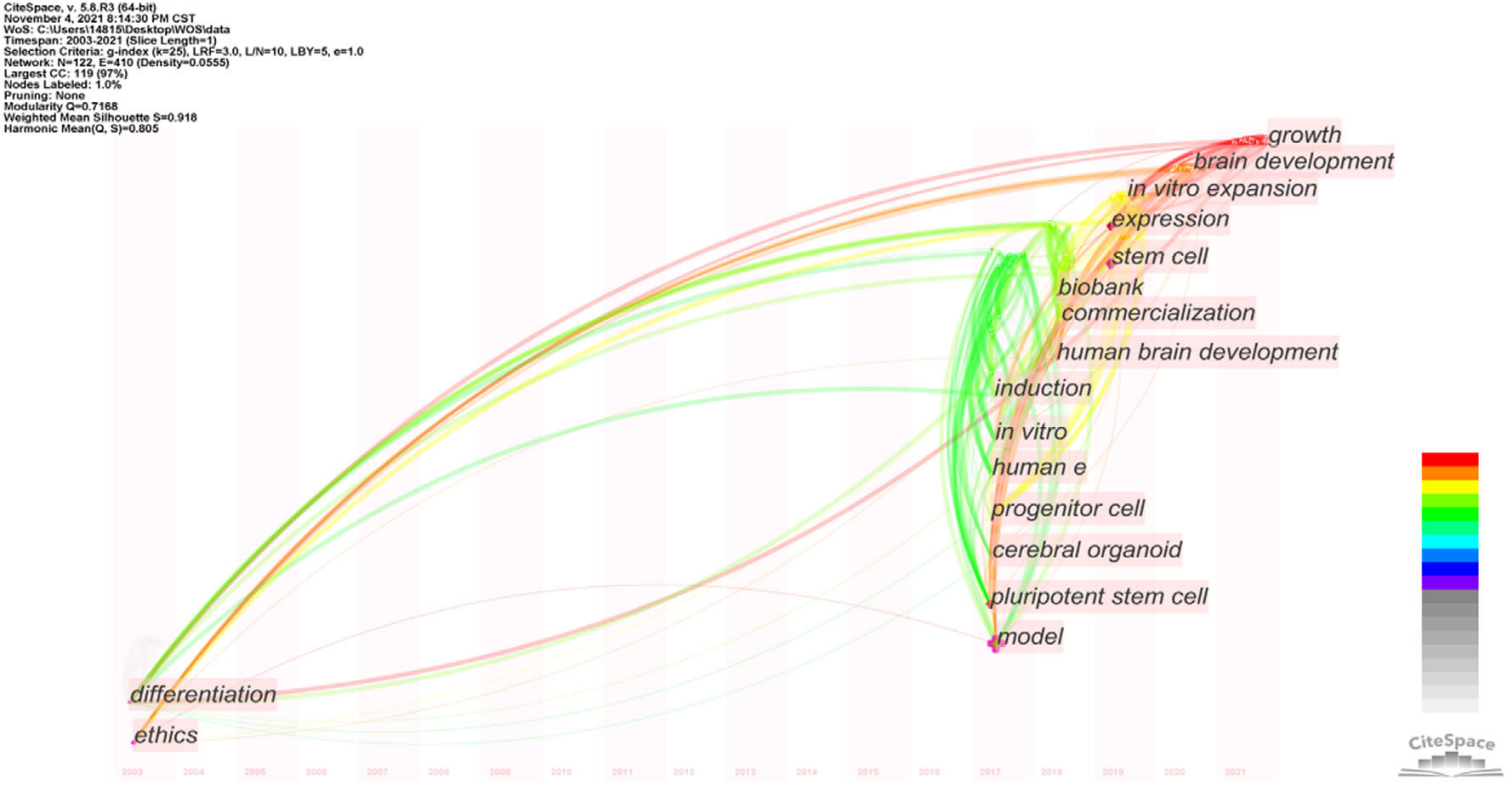
Keyword time‐zone graph of the research on ethical concerns of cerebral organoids

The research trend of ethical concerns of cerebral organoids is displayed in the keyword time zone graph (Figure 4), in which the keyword was used as the analysis node, and the ‘Timezone View’ display mode was selected to formulate the network graph to obtain a time‐series distribution diagram of domain keywords. We analysed the evolution of popular keywords from the time dimension based on the frequency of appearance, year of first appearance, and PageRank index and identified the research trends and features of this field. If a new keyword co‐occurs with the keyword after the year of its first appearance, it is connected to the time zone in which the new keyword is located with a line, indicating the inheritance and evolutionary relationship of the research topic. More connecting lines indicate a keyword's higher PageRank index, and thicker connection lines indicate a higher continuity of the keyword. The larger the connection span is, the longer the keyword inheritance time is.

As illustrated in Figures 1 and 4, research on the ethical concerns of cerebral organoids has increased immensely since 2017. The research is primarily divided into two periods. Keywords before 2017 included ‘differentiation’ and ‘ethics’ and keywords after 2017 primarily included ‘model’, ‘pluripotent stem cell’, ‘cerebral organoid’, progenitor cell’, ‘humane e’, ‘in vitro’, ‘induction’, ‘human brain development’, ‘commercialization’, ‘biobank’, ‘stem cell’, ‘expression’, ‘in vitro expansion’, ‘brain development’ and ‘growth’. This indicates that most of the keywords are relatively new in the field. In contrast to the first period, most of the 15 keywords used since 2017 are related to each other, and some of them are related to the keywords from the first time period. The keywords ‘ethics’ and ‘brain development’ cover a large time span, and the connection line between them is thick, which indicates that this group of keywords has a high frequency of joint occurrence, excellent continuity and a close evolutionary relationship. This also demonstrates that the research on ethics in this field has a long history.

As indicated in Figure 4, academics began increasingly focusing on ethical concerns in 2017. Through our analysis, we determined that such attention was intrinsically related to the continual development and breakthrough of technology after 2016. For instance, in 2017, *Nature Methods* awarded the annual technology award to organoid technology,^40^ and a research group reported that the neurons in cerebral organoids discharge electricity when photosensitive cells in the cerebral organoids' retinas are exposed to light.^41^ In 2018, researchers claimed that grafted organoids were integrated into a mouse's brain,^42^ and in 2019, researchers claimed that the coordinated activity waves generated by cerebral organoids are similar to those observed in preterm human electroencephalography.^43^ In 2020, a research team used cerebral organoid technologies to reveal the connection between the apolipoprotein E genotype and Alzheimer disease.^44^ In 2021, a study demonstrated that the development of cerebral organoids continues beyond the fetal stage.^45^

This series of technological breakthroughs is reflected in the graph in Figure 4. Many keywords that initially emerged were related to technology, but as technology continued to advance, particularly after 2016 when cerebral organoids were gradually mimicking the human brain more closely and increasingly being combined with chimera research, ethical concerns attracted more attention. Thus, the keyword ‘ethics’ has appeared in association with emerging keywords since 2017, particularly in conjunction with ‘brain development’.

## 7 THE FUTURE

Based on the keyword time zone graph, literature data analysis and clustering topic analysis of studies on ethical concerns of cerebral organoids, several future research hotspots can be predicted.

Although researchers maintain that existing cerebral organoids are too primitive to generate consciousness, the consciousness of future advanced cerebral organoids depends on the definition of consciousness.^42^ However, a clear, unified and operable definition of consciousness does not exist, neither do standards or methods for assessing consciousness. In October 2019, a conference held at the University of California, San Diego, was delayed by several months for the reason that participants could not agree on the definition of consciousness.^46^ Some scholars rank an individual's degree of consciousness in ascending order: conscious access to sensory stimulation, wakefulness, vigilance, focal attention, sentience and subjective self‐awareness.^19^ Some experts believe that complex forms of consciousness can only be attained in certain social contexts and through language acquisition; thus, human cerebral organoids in a petri dish can never develop actual consciousness.^25^ The definition, assessment methods and standard of consciousness; attainment of true consciousness by human cerebral organoids; and other relevant ethical concerns are the first vital concerns that should be addressed in the future.

Cerebral organoid technologies are combined with other life science technologies (e.g., chimeras and brain–computer interfaces). For instance, in 2018, Gage's team at the Salk Institute for Biological Studies claimed to have cultured human cerebral organoids for 40 to 50 days in a petri dish, and to further, they inserted the organoids into cavities crafted at Retrosplenial Cortex of an adult mouse, which is a crucial area for motor and space acquisition. Then on day 14, dense rete vasculosums have formed within the organoids, as concentrations of certain markers of the organoids suggested the development into neurons from human neural progenitor cells, and forming synapses. On day 90 after the transplantation, those human cerebral organoids in mice brain had generated axons.^42^ Human–animal chimeras with cerebral organoids have been produced to conduct enhanced research, and more cross‐field research on cerebral organoids and brain–computer interfaces now exists, which will likely lead to additional ethical debates. Therefore, future ethical risks related to these cross‐fields will be a vital topic of future research.

Lastly, the governance of ethical concerns related to cerebral organoids will be attributed to the formulation of laws and regulations. No relevant global laws or regulations exist specifically for cerebral organoids. Australia has regulations related to organoid research,^30^ and the National Health and Medical Research Council's National Statement on Ethical Conduct in Human Research clarifies the donor's right to informed consent and the participant's right to know the research results. Although the statement addresses the welfare of donors, it does not reflect the unique ethical concerns of cerebral organoid research.^30^ Similarly, regulations in the United States on cerebral organoid research do not solve the ethical concerns specific to the consciousness and moral status of cerebral organoids. Therefore, legal formulation and the supervision of ethical concerns related to cerebral organoids is another future research hotspot.

## 8 CONCLUSIONS

Although research on cerebral organoids can benefit the biomedicine field, various ethical concerns emerge as this technology advances. Knowledge graphs and statistical analysis revealed that although the ethical concerns of cerebral organoids have long been discussed, ethical concerns have only just begun to receive increasing attention. In the process of constructing the knowledge graphs, only more than one hundred related literatures were found. Since 2017, the number of the published articles on the field has increased, which is closely related to technological breakthroughs. Through clustering topic analysis, it is found that the ethical concerns are primarily divided into those that are common in life sciences, specific to brain organs, and appear across domains. These increasing ethical concerns are inherently related to the continual development of technology after 2016, particularly the development of pluripotent stem cells, the 3D culture system and the cross‐application of cerebral organoid technology and other technologies (e.g., chimeras and brain–computer interfaces). Future research should focus on the ethical concerns of consciousness that are unique to cerebral organoids, ethical concerns of cross‐fields and construction and improvement of legislative and regulatory systems. By promoting the study and discussion of the ethical concerns in this field prospectively, relevant problems will be solved, and the healthy development of this field will be promoted.

## AUTHOR CONTRIBUTIONS

Lulu Ding and Zhenyu Xiao are co‐first authors of the article, and they contributed to this work equally. Lulu Ding and Zhenyu Xiao was involved in the collection and/or assembly of data, data analysis and interpretation, and manuscript writing. Xia Gong was involved in the collection and/or assembly of data. Yaojin Peng proposed the conception, designed the experiments and research, and was involved in financial support, collection and/or assembly of data, data analysis and interpretation, manuscript writing, and final approval of the manuscript.

## Data Availability

The data used to support the findings of this study are available from the corresponding author upon request.
